# COF-SiO_2_@Fe_3_O_4_ Composite for Magnetic Solid-Phase Extraction of Pyrethroid Pesticides in Vegetables

**DOI:** 10.3390/molecules29102311

**Published:** 2024-05-14

**Authors:** Ling Yu, Aiqing Xia, Yongchao Hao, Weitao Li, Xu He, Cuijuan Xing, Zan Shang, Yiwei Zhang

**Affiliations:** 1 College of Chemistry and Chemical Engineering, Xingtai University, Xingtai 054001, China; xiaaiqing59420@sina.com (A.X.); haoyongchao2015@163.com (Y.H.); liweitao2021xtxy@163.com (W.L.); hexunew@outlook.com (X.H.); s15530181703@163.com (Z.S.); zyw17659928836@163.com (Y.Z.); 2 Functional Polymer Materials R&D and Engineering Application Technology Innovation Center of Hebei, Xingtai 054001, China

**Keywords:** covalent organic framework, magnetic solid-phase extraction, gas chromatography–mass spectrometry, pyrethroid pesticides

## Abstract

Pyrethroid pesticides (PYRs) have found widespread application in agriculture for the protection of fruit and vegetable crops. Nonetheless, excessive usage or improper application may allow the residues to exceed the safe limits and pose a threat to consumer safety. Thus, there is an urgent need to develop efficient technologies for the elimination or trace detection of PYRs from vegetables. Here, a simple and efficient magnetic solid-phase extraction (MSPE) strategy was developed for the simultaneous purification and enrichment of five PYRs in vegetables, employing the magnetic covalent organic framework nanomaterial COF-SiO_2_@Fe_3_O_4_ as an adsorbent. COF-SiO_2_@Fe_3_O_4_ was prepared by a straightforward solvothermal method, using Fe_3_O_4_ as a magnetic core and benzidine and 3,3,5,5-tetraaldehyde biphenyl as the two building units. COF-SiO_2_@Fe_3_O_4_ could effectively capture the targeted PYRs by virtue of its abundant π-electron system and hydroxyl groups. The impact of various experimental parameters on the extraction efficiency was investigated to optimize the MSPE conditions, including the adsorbent amount, extraction time, elution solvent type and elution time. Subsequently, method validation was conducted under the optimal conditions in conjunction with gas chromatography–mass spectrometry (GC-MS). Within the range of 5.00–100 μg·kg^−1^ (1.00–100 μg·kg^−1^ for bifenthrin and 2.5–100 μg·kg^−1^ for fenpropathrin), the five PYRs exhibited a strong linear relationship, with determination coefficients ranging from 0.9990 to 0.9997. The limits of detection (LODs) were 0.3–1.5 μg·kg^−1^, and the limits of quantification (LOQs) were 0.9–4.5 μg·kg^−1^. The recoveries were 80.2–116.7% with relative standard deviations (RSDs) below 7.0%. Finally, COF-SiO_2_@Fe_3_O_4_, NH_2_-SiO_2_@Fe_3_O_4_ and Fe_3_O_4_ were compared as MSPE adsorbents for PYRs. The results indicated that COF-SiO_2_@Fe_3_O_4_ was an efficient and rapid selective adsorbent for PYRs. This method holds promise for the determination of PYRs in real samples.

## 1. Introduction

Pyrethroid pesticides (PYRs), originating the 1970s, emerged as a class of synthetic insecticides acclaimed for their affordability, broad insecticidal spectrum, minimal toxicity, low residue levels and eco-friendly nature. They significantly contributed to pest management and found extensive application in vegetables, fruits and other agricultural products [1]. However, mounting evidence indicates that PYRs could potentially act as endocrine disruptors, compromising animals’ endocrine function and exerting estrogenic effects on the environment [2]. Toxic substances can kill embryos prior to and after implantation or cause malformation in various organs [3]. Prolonged exposure to PYRs and their metabolites may result in endocrine-disrupting effects and sublethal toxicity. With the increasing awareness of food safety, several organizations have established maximum residue limits (MRLs) for PYRs in fruits and vegetables, such as 0.01–0.5 mg·kg^−1^ in the European Union [4] and 0.01–10 mg·kg^−1^ in China (GB 2763-2021). Consequently, it is crucial to develop a straightforward, efficient and highly sensitive method for the preconcentration and determination of trace levels of PYRs in complex vegetable matrices.

The matrices of vegetable samples contain large amounts of pigments, cellulose and minerals, which could dramatically hinder the detection of trace PYRs in food. Therefore, it is imperative to efficiently enrich and purify multiple PYRs in vegetable samples prior to instrumental analysis. Numerous sample pretreatment methods have been documented for PYR analysis, such as liquid–liquid extraction (LLE) [5], QuEChERS methods [6,7], solid-phase extraction (SPE) [8], dispersed liquid–liquid microextraction (DLLME) [9], stir bar sorptive extraction (SBSE) [10] and solid-phase microextraction (SPME) [11]. Magnetic solid-phase extraction (MSPE) has the advantages of easy separation, convenient operation and time-saving qualities [12,13]. In the MSPE process, magnetic sorbents are directly dispersed in the sample solution for the rapid and efficient extraction of analytes and then quickly separated by an external magnetic field. Currently, MSPE is widely used in the fields of environmental governance [14,15], biotechnology [16,17], medicine [18] and food [19,20]. To the best of our knowledge, the selection of adsorbents for target analyte-based MSPE procedures is one of the most important factors in efficient extraction and separation. An enhanced sensitivity and cleanup strategy for PYR determination using functionalized nanomaterials in an adsorbent-based MSPE procedure was previously reported for different samples, such as mesoporous composite Fe_3_O_4_@SiO_2_@KIT-6 [21], magnetic silica aerogels [22], polystyrene-coated magnetic nanoparticles [23] and metal–organic frameworks such as Fe_3_O_4_@TMU-21 [24], etc. In this regard, researchers have recently focused on developing new adsorbents or nanoparticles with certain selectivity, high extraction efficiency and anti-interference abilities for PYR determination in complex food and environmental analysis.

A covalent organic framework (COF) is a new type of porous polymer material that can be constructed with organic building units via covalent bonds of elements (C, O, N, H, etc.) [25]. The structure and surface properties are predominantly dependent on covalently linked topological schemes and organic monomers. They exhibit exceptional characteristics, including an adjustable pore size and high chemical stability, rendering them highly promising for applications in catalysis [26,27], sensing [28], optoelectronic devices [29] and separation [30,31]. However, the challenge of separating COFs from a solution due to their low density impedes their widespread application in separation and enrichment. Fortunately, the combination of COFs with Fe_3_O_4_ provides a potential solution to this issue, mitigating the substantial loss of COFs and facilitating their practical utilization.

**Scheme 1:**
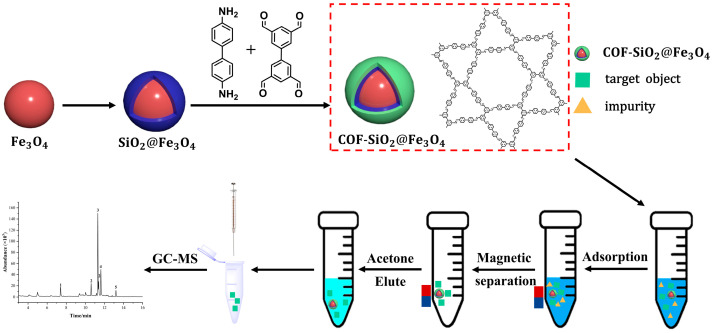
Synthesis of COF-SiO_2_@Fe_3_O_4_ and determination of PYRs by MSPE.

During this study, a new material, COF-SiO_2_@Fe_3_O_4_, was successfully synthesized under solvothermal conditions. Employing it as an extractant, in conjunction with GC-MS, a sample pretreatment method for the MSPE of trace amounts of PYRs in vegetables was established. The synthesized COF-SiO_2_@Fe_3_O_4_ demonstrated advantages such as excellent thermal stability, rapid separation ability and high selectivity for PYRs. Simultaneously, the results of different adsorbents used in MSPE for the detection of PYRs in vegetables proved that, compared with Fe_3_O_4_ and NH_2_-SiO_2_@Fe_3_O_4_, COF-SiO_2_@Fe_3_O_4_ showed the highest adsorption capacity on PYRs. This superiority can be attributed to the abundant π- electron system and enriched hydroxyl groups on COF-SiO_2_@Fe_3_O_4_’s surface. These features make it a satisfactory adsorbent for PYRs through π–π interactions and hydrogen bonding between the adsorbent surface and pesticide molecules. Furthermore, COF-SiO_2_@Fe_3_O_4_ was successfully applied for the excellent adsorption of PYRs in vegetable samples. Scheme 1 provides a schematic diagram detailing the synthesis of COF-SiO_2_@Fe_3_O_4_ and the determination of PYRs via MSPE.

## 2. Results and Discussion

### 2.1. Characterization of COF-SiO_2_@Fe_3_O_4_

**Figure 1:**
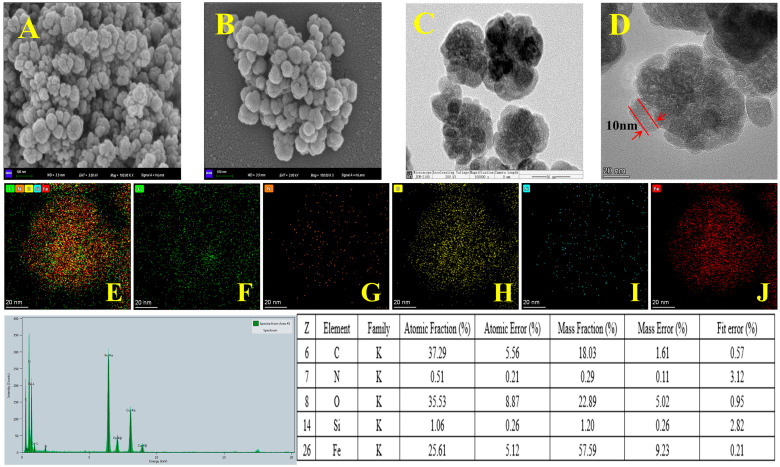
SEM images of Fe_3_O_4_ (**A**) and COF-SiO_2_@Fe_3_O_4_ (**B**), TEM images of COF-SiO_2_@Fe_3_O_4_ (**C**,**D**) and EDS spectra of COF-SiO_2_@Fe_3_O_4_ (**E**–**J**).

The surface morphology of the prepared materials was characterized by SEM and TEM. The SEM images of Fe_3_O_4_ and COF-SiO_2_@Fe_3_O_4_ and the TEM images of COF-SiO_2_@Fe_3_O_4_ are shown in Figure 1. It can be seen that the Fe_3_O_4_ nanoparticles present a spherical structure with a rough surface (Figure 1A). Upon modification with the COF, a relatively smooth surface was observed in the SEM image of COF-SiO_2_@Fe_3_O_4_ (Figure 1B). The TEM images showed that the average particle size of COF-SiO_2_@Fe_3_O_4_ was approximately 100 nm, with the COF shell thickness measuring approximately 10 nm (Figure 1D). Furthermore, EDS was conducted to identify the elements present in COF-SiO_2_@Fe_3_O_4_ (Figure 1E–G). It can be seen that elements Fe, C, N, Si and O were uniformly distributed in the material, confirming the successful synthesis of COF-SiO_2_@Fe_3_O_4_. Notably, among them, the content of Fe, C and O elements constituted the majority, accounting for approximately 25%, 37% and 35%, respectively. The presence of the C element primarily stemmed from the synthesis of covalent organic frameworks, while the N element was mainly derived from the ligand benzidine. The TEM and EDS images support the conclusion that COF-SiO_2_@Fe_3_O_4_ comprises a thin layer of COFs enveloping the Fe_3_O_4_ nanoparticles.

**Figure 2:**
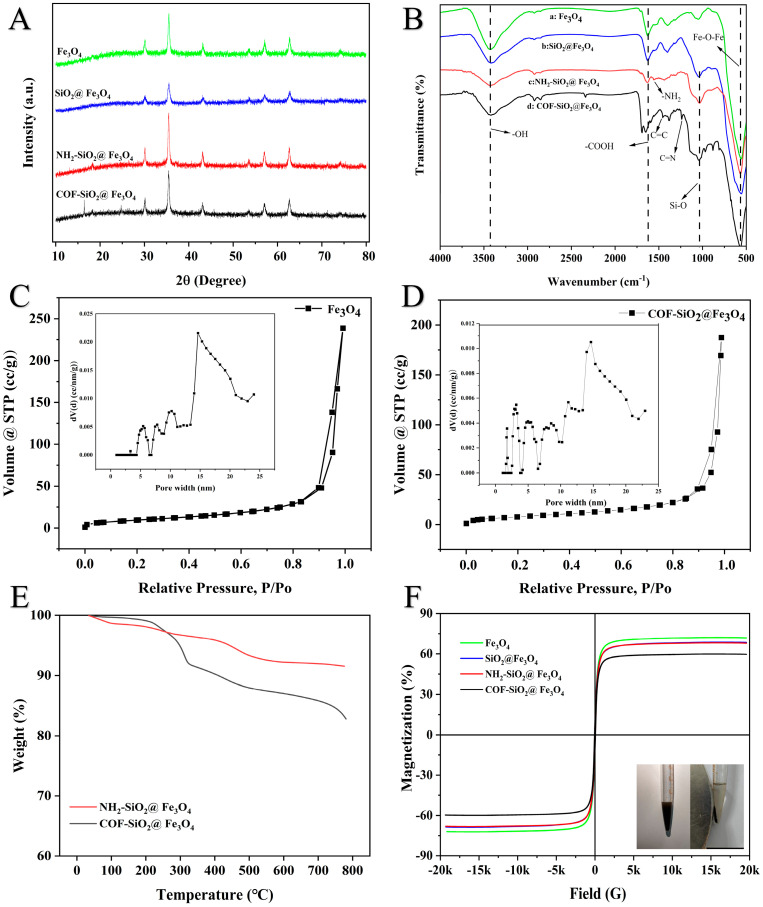
XRD patterns of Fe_3_O_4_, SiO_2_@Fe_3_O_4,_ NH_2_-SiO_2_@Fe_3_O_4_ and COF-SiO_2_@Fe_3_O_4_ (**A**); FTIR spectra of Fe_3_O_4_ (**B**−a), SiO_2_@Fe_3_O_4_ (**B**−b), NH_2_-SiO_2_@Fe_3_O_4_ (**B**−c) and COF-SiO_2_@Fe_3_O_4_ (**B**−d); N_2_ adsorption–desorption isotherms of Fe_3_O_4_ and COF-SiO_2_@Fe_3_O_4_ (**C**,**D**); TGA spectra of NH_2_-SiO_2_@Fe_3_O_4_ and COF-SiO_2_@Fe_3_O_4_ (**E**); and magnetic hysteresis curves of Fe_3_O_4_, SiO_2_@Fe_3_O_4_, NH_2_-SiO_2_@Fe_3_O_4_ and COF-SiO_2_@Fe_3_O_4_ (**F**).

The XRD patterns of the Fe_3_O_4_, SiO_2_@Fe_3_O_4_, NH_2_-SiO_2_@Fe_3_O_4_ and COF-SiO_2_@Fe_3_O_4_ materials are shown in Figure 2A. Characteristic diffraction peaks could be observed at 2θ = 30.1° (200), 35.5° (311), 43.5° (400), 53.7° (422), 57.2° (511) and 62.5° (440), which were all attributed to the magnetic center body, indicating that the synthesized material had a good crystal structure [32]. In the XRD pattern of SiO_2_@Fe_3_O_4_, there were no other diffraction peaks that emerged, indicating that the coated SiO_2_ shell was amorphous. Additionally, the observed diffraction peaks of COF-SiO_2_@Fe_3_O_4_ at 2θ were 16.8° and 24.5°, corresponding to (120) and (001), respectively [33], confirming the successful formation of the COF-SiO_2_@Fe_3_O_4_ nanocomposite.

The FTIR spectra of Fe_3_O_4_, SiO_2_@Fe_3_O_4_, NH_2_-SiO_2_@Fe_3_O_4_ and COF-SiO_2_@Fe_3_O_4_ (4000–500 cm^−1^) are depicted in Figure 2B. The characteristic peak at 3440 cm^−1^ corresponds to the stretching vibration peak of the -OH group. Additionally, the typical band at 577 cm^−1^ was assigned to the Fe-O-Fe vibration, providing evidence for the presence of Fe_3_O_4_. Furthermore, the characteristic band at 1640 cm^−1^ indicated the presence of carboxyl groups (curve a) [34]. The peak at 1080 cm^−1^ represents the tensile vibration of the Si-O group (curve b), signifying the successful loading of SiO_2_ onto the particle surface. Following the amination modification of Fe_3_O_4_@SiO_2_, a new peak emerges at 1560 cm^−1^ (curve c), corresponding to -NH_2_ on the surfaces of the SiO_2_ nanoparticles. The peak disappears after the formation of COF-SiO_2_@Fe_3_O_4_, replaced by new peaks at 1250 cm^−1^ and 1480 cm^−1^, corresponding to C=N and aromatic C=C groups, respectively (curve d). This observation indicates the successful attachment of the COFs to the magnetic nanoparticle surface. The surface functional groups (–NH_2_, –OH, C=N) of COF-SiO_2_@Fe_3_O_4_ establish a foundation for hydrogen bonding with the analyte PYRs.

Figure 2C,D illustrate the N_2_ adsorption–desorption isotherms and the pore size distribution curves of the COF-SiO_2_@Fe_3_O_4_ nanocomposites. As displayed in Figure 2C,D, the isotherms of both materials resemble type IV isotherms and H3-type hysteresis loops according to the IUPAC classification, indicating the predominantly mesoporous structure of the materials. The calculated BET surface area and pore volume of the Fe_3_O_4_@COF nanocomposites were 30.09 m^2^⋅g^−1^ and 0.29 cm^3^⋅g^−1^, respectively, while those of the bare Fe_3_O_4_ nanoparticles were 42.39 m^2^⋅g^−1^ and 0.37 cm^3^⋅g^−1^. The inset presents the pore size distribution, revealing a multistage porous structure. The mesopore sizes were primarily centered around 5.0, 8.5, 11.1 and 14.6 nm in the Fe_3_O_4_ nanoparticles. Notably, COF-SiO_2_@Fe_3_O_4_ displayed two additional peaks centered at 1.7 and 3.1 nm, which were similar to the previously reported pore diameter of the bulk COFs [35]. It indicated the increased presence of micropores compared to Fe_3_O_4_, potentially contributing to its excellent selectivity.

The mass ratios of different components and the thermal stability of the COF-SiO_2_@Fe_3_O_4_ nanocomposites were examined by TGA. As depicted in Figure 2E, NH_2_-SiO_2_@Fe_3_O_4_ exhibited 3.2 wt% loss in the temperature range of 100–300 °C, attributed to the weight loss of the absorbed water [34]. Conversely, the COF-SiO_2_@Fe_3_O_4_ nanocomposites displayed a notable weight loss of approximately 8.5 wt% beyond 360 °C, implying the presence of COFs on the surfaces of the Fe_3_O_4_ nanoparticles. This observation aligns with the thin COF layer evident in the SEM, TEM and EDS images. Furthermore, the COF-SiO_2_@Fe_3_O_4_ nanocomposites demonstrated excellent thermal stability up to 220 °C. In addition, the COF-SiO_2_@Fe_3_O_4_ nanocomposites were immersed in water and different solvents, such as THF, DMSO, methanol and acetone, for 24 h to examine the chemical stability. The results demonstrated that all products presented the same adsorption peaks in the FTIR spectra (Figure S1, Supplementary Information), indicating the good chemical stability of the COF-SiO_2_@Fe_3_O_4_ nanocomposites.

The saturation magnetization values of the Fe_3_O_4_, SiO_2_@Fe_3_O_4_, NH_2_-SiO_2_@Fe_3_O_4_ and COF-SiO_2_@Fe_3_O_4_ nanocomposites were measured at 71.85, 68.67, 67.97 and 59.59 emu⋅g^−1^, respectively. The hysteresis curves of all magnetic nanoparticles exhibited the S type, indicating their superparamagnetic characteristics (Figure 2F). Among them, the magnetization of COF-SiO_2_@Fe_3_O_4_ was the lowest, being 12.26 emu⋅g^−1^ lower than that of the Fe_3_O_4_ nanoparticles, attributed to the magnetism reduction caused by the COF wrapping around Fe_3_O_4_. Despite this, the high saturation magnetism of the COF-SiO_2_@Fe_3_O_4_ nanocomposites proved sufficient for magnetic separation. As displayed in the inset of Figure 2F, the COF-SiO_2_@Fe_3_O_4_ nanocomposites, when homogeneously dispersed in an aqueous solution, could be rapidly gathered within 0.5 min with the assistance of an external magnet, resulting in the immediate clarification of the solution. Hence, the incorporation of Fe_3_O_4_ not only imparts excellent permanent magnetic properties to the COF but also maintains its outstanding physical and chemical properties.

### 2.2. Optimization of MSPE Parameters

To obtain higher extraction recoveries, various parameters influencing the extraction efficiency of MSPE were investigated, such as the amount of adsorbent, extraction time, elution solvent and elution time. All experiments were repeated in triplicate, with solutions spiked with analytes at a concentration of 50 μg·kg^−1^. Single-factor experimentation was employed to optimize the data, with the initial experimental conditions as follows: 10 mg of adsorbent, an extraction time of 10 min, acetone as the elution solvent and an elution time of 5 min. Subsequently, upon determining a single-factor variable, the next variable was explored, building upon the established optimized parameter.

#### 2.2.1. Effect of Adsorbent Amount

**Figure 3:**
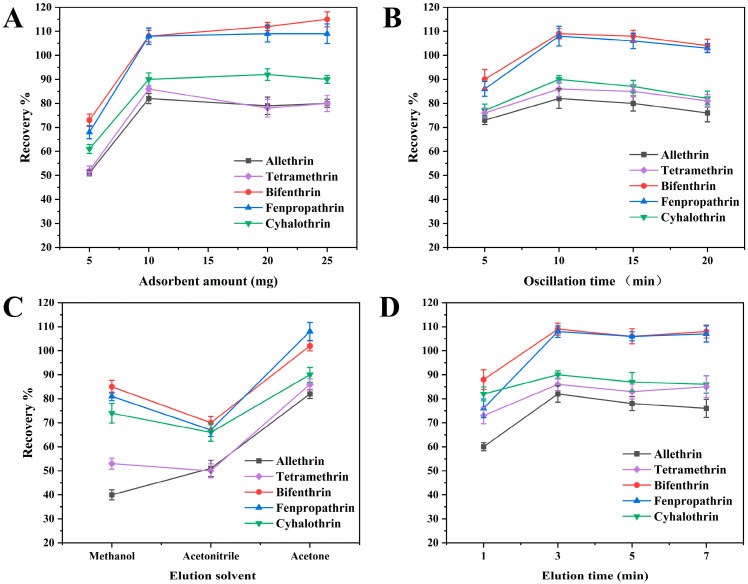
Optimization of adsorbent amount (**A**), extraction time (**B**), elution solvent (**C**) and elution time (**D**).

The amount of adsorbent typically has a significant direct effect on the recovery of PYRs. To determine the optimum amount of the adsorbent, various masses of COF-SiO_2_@Fe_3_O_4_ ranging from 5 to 25 mg were evaluated. As depicted in Figure 3A, the recoveries exhibited a significant improvement when the adsorbent amount was increased from 5 to 10 mg. However, further increments in the adsorbent amount did not enhance the extraction efficiency of the five PYRs. Consequently, in order to improve the recoveries and reduce the amount of adsorbent, 10 mg of COF-SiO_2_@Fe_3_O_4_ was found to be optimal and was used as the adsorbent in subsequent experiments.

#### 2.2.2. Effect of Extraction Time

The adsorption time is one of the most effective factors during the MSPE process. The method of shaking can increase the contact area between the adsorbent and analytes and then accelerate the adsorption of the analytes onto the adsorbent. The effects of different shaking times (5–20 min) on the extraction efficiencies were investigated by keeping the other experimental conditions constant. The results were demonstrated in Figure 3B. The adsorption efficiencies of the PYRs increased with the adsorption time and reached an adsorption equilibrium at 10 min. Further prolonging the time did not increase the adsorption efficiencies of the analytes. It was expected that as the adsorption increased, the recovery of the target ions would increase. When the adsorption equilibrium was reached, all active sites of the prepared nanocomposite were closed. It caused the desorption of some of the adsorbed elements, resulting in reduced recoveries. Hence, 10 min was selected as the optimal adsorption time.

#### 2.2.3. Effect of Elution Solvent

The selection of an appropriate elution solvent is crucial in efficiently desorbing the target analytes from the extracted adsorbents. Here, acetonitrile, methanol and acetone were used as elution solvents to evaluate their elution performance. As depicted in Figure 3C, acetone exhibited significantly improved the desorption efficiency of the target compounds as the elution solvent. Conversely, the recoveries of allethrin were low when methanol was used as the eluent solvent, while the recoveries of the five PYRs were generally low when acetonitrile was employed as an eluent solvent. Hence, acetone was selected as the optimal elution solvent for subsequent experiments.

#### 2.2.4. Effect of Elution Time

The effect of the elution time was investigated using ultrasound for durations of 1, 3, 5 and 7 min, with the results presented in Figure 3D. Increasing the time from 1 to 3 min led to the recoveries rising by 80–109%. Subsequently, extending the elution time from 3 to 5 min did not notably alter the recoveries of the PYRs, as the adsorption and desorption equilibrium had been reached at 3 min. However, with a further increase in the ultrasonic time from 5 to 7 min, there was a slight improvement in the PYR recoveries. This can be attributed to the release of heat during ultrasonic treatment, causing the organic solvent to vaporize and thereby increasing the concentration of PYRs in acetone. Consequently, 3 min was determined to be the optimal elution time.

### 2.3. Method Validation

The quantitative analysis of the five PYRs was further evaluated using COF-SiO_2_@Fe_3_O_4_-based MSPE coupled with GC-MS. Under the optimized conditions, method validations were also conducted, encompassing the linearity, limits of detection (LODs, S/N = 3), limits of quantification (LOQs, S/N = 10), enrichment factors (EFs) and reproducibility, as summarized in Table 1. The developed method exhibited excellent linearity, with correlation coefficients (r) exceeding 0.9990 within the range of 5–100 μg·kg^−1^ (1.00–100 μg·kg^−1^ for bifenthrin and 2.5–100 μg·kg^−1^ for fenpropathrin, respectively). The LODs for the five PYRs ranged from 0.3 to 1.5 μg·kg^−1^, with the corresponding LOQs found to be 0.9–4.5 μg·kg^−1^. The EFs of PYRs, defined as the ratio of the analyte concentration in the extract to that in the original sample, ranged from 4.4 to 12.4. The inter-day RSDs were obtained by extracting a standard solution five times within a day, and the intra-day RSDs were determined by extracting a standard solution that had been independently prepared for six consecutive days. The inter- and intra-day RSDs were in the range of 1.9–6.2% and 2.3–7.0%, respectively, indicating acceptable reproducibility. Additionally, the reproducibility of the COF-SiO_2_@Fe_3_O_4_ nanocomposites was assessed by the batch-to-batch RSDs. The sample solution contained 50 μg·kg^−1^ of each analyte. The results revealed batch-to-batch RSDs of less than 4.2%, suggesting the good synthetic reproducibility of COF-SiO_2_@Fe_3_O_4_.

### 2.4. Real Sample Analysis

To evaluate the reliability and feasibility of the developed method, cucumber, cabbage and lettuce samples were collected for PYR extraction and determination. Each sample underwent five repeated analyses. The results showed that none of the PYRs were detected in the vegetables. To further verify the method developed in actual samples, vegetable samples spiked with different concentrations of PYRs were analyzed. The three vegetables were spiked with PYR standards at concentrations of 5, 10 and 20 μg⋅kg^−1^ for low, medium and high levels, respectively. The subsequent extraction procedure was conducted, with the RSDs (n = 3) and average recoveries given in Table 2. The recoveries of the five PYRs ranged from 80.2 to 116.5% with RSDs of 2.3–6.7% for cucumber, 81.7–114.7% with RSDs of 2.1–6.8% for cabbage and 81.6–116.7% with RSDs of 2.4–7.0% for lettuce. The results indicate that the proposed MPSE-GC-MS method is effective for the enrichment and determination of PYRs in vegetable samples.

### 2.5. Comparison of COF-SiO_2_@Fe_3_O_4_, NH_2_-SiO_2_@Fe_3_O_4_ and Fe_3_O_4_ as MSPE Adsorbents

**Figure 4:**
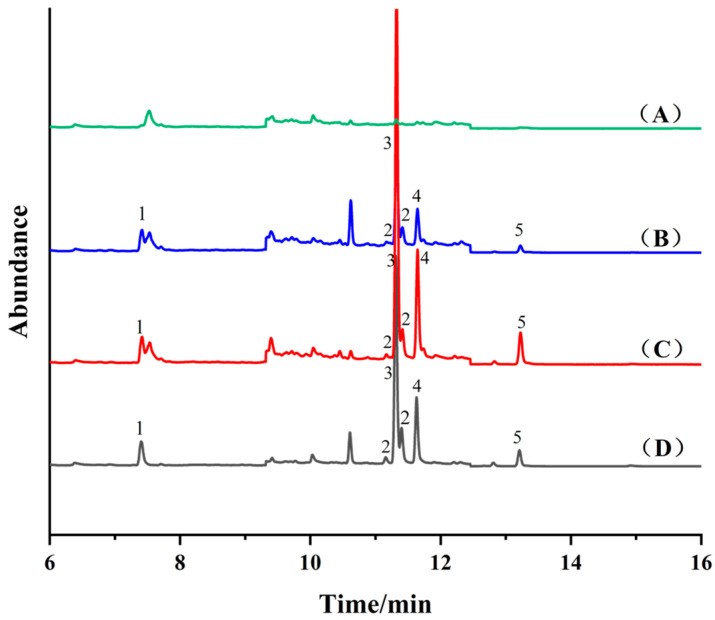
Total ion flow chromatography of blank cabbage sample (**A**) and spiked cabbage sample (50 μg⋅kg^−1^) after purifcation by Fe_3_O_4_ (**B**), NH_2_-SiO_2_@Fe_3_O_4_ (**C**), COF-SiO_2_@Fe_3_O_4_ (**D**), respectively. 1—Allethrin, 2—Tetramethrin, 3—Bifenthrin, 4—Fenpropathrin, 5—Cyhalothrin.

Fe_3_O_4_ (10 mg), NH_2_-SiO_2_@Fe_3_O_4_ (10 mg) and COF-SiO_2_@Fe_3_O_4_ (10 mg) were used as adsorbents for the MSPE of a blank cabbage extract (50 μg·kg^−1^) with the standard addition of PYRs, followed by GC-MS detection, as shown in Figure 4. Notably, NH_2_-SiO_2_@Fe_3_O_4_ and COF-SiO_2_@Fe_3_O_4_ exhibited superior enrichment effects on PYRs compared to Fe_3_O_4_. When employing COF-SiO_2_@Fe_3_O_4_ as the MSPE adsorbent, fewer impurity peaks were observed in the chromatographic curve. Specifically, those interfering with the target allethrin were significantly reduced, thereby enhancing the accuracy of the method. COF-SiO_2_@Fe_3_O_4_ demonstrated remarkable performance, such as certain selectivity, high extraction efficiency and anti-interference abilities regarding PYRs, affected by their respective structures. An analysis of the structural formulas of the PYRs (Table S1) revealed their abundant benzene rings, facilitating strong π–π stacking interactions with the synthesized COF-SiO2@Fe_3_O_4_ due to their π–π conjugation [36]. Furthermore, the surface functional groups (–NH_2_, –OH, C=N) of COF-SiO_2_@Fe_3_O_4_ and PYRs exhibit strong hydrogen bond interactions [37], which was confirmed by the FTIR spectra results.

### 2.6. Comparison with Other References

Table 3 compares the results for the extraction of pesticides for the reported method and other common methods. The precision of the MSPE/GC-MS using COF-SiO_2_@Fe_3_O_4_ as a sorbent was comparable to that of other methods [9,10,11,37,38]. The LODs achieved in this work are higher than the those obtained using other methods, yet they still satisfy the detection standard for PYRs in vegetables. Moreover, the proposed method requires a shorter extraction time compared to other methods.

## 3. Experimental Section

### 3.1. Materials and Chemicals

Benzidine (95%) and 3,3,5,5-tetraaldehyde biphenyl (97%) were purchased from Shanghai Aladdin Biochemical Technology Co., Ltd. (Shanghai, China). Allethrin, tetramethrin, bifenthrin, fenpropathrin and cyhalothrin were obtained from Beijing Putian Tongchuang Biological Technology Co., Ltd. (Beijing, China). FeCl_3_.6H_2_O and dimethyl sulfoxide (DMSO) were purchased from Tianjin Kemiou Chemical Reagent Co., Ltd. (Tianjin, China). Anhydrous sodium acetate, ethylene glycol, anhydrous ethanol, tetrahydrofuran (THF), toluene, tetraethoxysilane (TEOS), 3-aminopropyltriethoxysilane (APTES) and dimethyl silicone oil were purchased from the Tianjin Damao Chemical Reagent Factory (Tianjin, China). Chromatographic-grade acetonitrile was obtained from Shanghai Aladdin Biochemical Technology Co., Ltd. (Shanghai, China). Glacial acetic acid was purchased from the Tianjin Fuchen Chemical Reagent Factory (Tianjin, China). Ammonia water was purchased from Yantai Far East Fine Chemical Co., Ltd. (Yantai, China).

### 3.2. Equipment

GC/MS-QP 2010 Ultra (Shimadzu Corporation, Kyoto, Japan), SC-3612 centrifuge (Anhui Zhongke Zhongjia Scientific Instrument Co., Ltd., Hefei, China), SN-QX-20D ultrasonic cleaning machine (Shanghai Shangdun Instrument Equipment Co., Ltd., Shanghai, China), 2K-82B vacuum drying oven (Shanghai Instrument Experimental Factory, Shanghai, China), DHG-9023A blast drying oven (Shanghai Yiheng Scientific Instrument Co., Ltd., Shanghai, China), SHB-III circulating water multi-purpose vacuum pump (Zhengzhou Great Wall Technology & Trade Co., Ltd., Zhengzhou, China), RE-52AA rotary evaporator (Shanghai Yarong Biochemical Instrument Factory, Shanghai, China), YTGT-12 dry nitrogen blowing instrument (Shanghai Night Extension Technology Co., Ltd., Shanghai, China), Milli-Q ultrapure water apparatus (Millipore Corporation, Massachusetts, USA).

Scanning electron microscopy (SEM) images were obtained with a scanning electron microscope (GeminiSEM 300, ZEISS, Oberkochen, Germany). Transmission electron microscopy (TEM) images were recorded by a transmission electron microscope (Talos F200X, Thermo Scientific, Waltham, MA, USA). The energy-dispersive spectrum (EDS) analysis was conducted using a Super-X Spectrometer (Waltham, MA, USA). X-ray diffraction (XRD) data were achieved by X-ray diffraction (3kw, Rigaku, Tokyo, Japan). Fourier-transform infrared spectroscopy (FT-IR) was performed on a Nicolet iS20 spectrometer (Thermo Fisher, Waltham, MA, USA). Brunauer–Emmett–Teller (BET) values were measured by using an automatic surface and porosity analyzer (Nova 4000e, Quantachrome, Fort Lauderdale, FL, USA). Thermogravimetric analysis (TGA) was performed using a thermogravimetric analyzer (TGA/DSC 3+, Mettler Toledo, Zurich, Switzerland). The magnetization curves were measured by a vibrating sample magnetometer (VSM) (7404, LakeShore, Columbus, OH, USA).

### 3.3. Preparation of Standard Solution

The stock standard solutions of the five PYRs were individually prepared at a concentration of 5 μg·mL^−1^ in acetone and then stored at 4 °C before use. The working standard solutions were obtained freshly before use by diluting the stock solution to the desired concentration (0.02, 0.04, 0.06, 0.08, 0.1, 0.2, 0.4 μg·mL^−1^).

### 3.4. Preparation of Magnetic Materials

#### 3.4.1. Synthesis of Fe_3_O_4_ Magnetic Nanoparticles

Monodisperse Fe_3_O_4_ magnetic nanoparticles (MNPs) were synthesized via the solvothermal method [40]. Briefly, FeCl_3_·6H_2_O (1.352 g) and anhydrous sodium acetate (3.6 g) were dissolved in ethylene glycol (40 mL). The obtained homogeneous yellow solution was transferred to an autoclave and heated to 200 °C for 8 h. After the reaction, the product was collected by a magnet and washed with ultrapure water and ethanol several times and then dried at 60 °C in a vacuum drying oven.

#### 3.4.2. Synthesis of SiO_2_@Fe_3_O_4_ Magnetic Nanospheres

Fe_3_O_4_ (1 g) was dispersed in a mixture of ethanol (120 mL), ultrapure water (30 mL) and concentrated ammonia (2.4 mL). After ultrasonic dispersion for 5 min, TEOS (0.5 mL) was added and the mixture was stirred for 12 h at room temperature. During the process, TEOS underwent hydrolysis in an alkaline environment, resulting in the formation of a silica layer containing -OH groups on the surface of Fe_3_O_4_. The obtained brown precipitates were collected by magnetic separation and washed three times with ultrapure water and ethanol. Finally, the resultant SiO_2_@Fe_3_O_4_ nanocomposites were dried in a vacuum at 60 °C.

#### 3.4.3. Synthesis of NH_2_-SiO_2_@Fe_3_O_4_ Magnetic Nanospheres

SiO_2_@Fe_3_O_4_ (1.00 g) was dispersed in toluene (100 mL). Then, APTES (silane coupling agent) (10 mL) was added. APTES bonded to the surfaces of the Fe_3_O_4_ nanoparticles, forming ammoniated NH_2_-SiO_2_@Fe_3_O_4_. The reaction mixture was refluxed at 110 °C for 8 h and then magnetically separated after natural cooling and washed four times with toluene and ethanol. Finally, the resultant NH_2_-SiO_2_@Fe_3_O_4_ nanocomposites were dried at 60 °C for further use.

#### 3.4.4. Synthesis of COF-SiO_2_@Fe_3_O_4_ Magnetic Nanoparticles

NH_2_-SiO_2_@Fe_3_O_4_ (0.2 g), benzidine (0.185 g) and 3,3,5,5-tetraaldehyde biphenyl (0.133 g) were added to 80 mL of DMSO and ultrasonically dispersed for 10 min to form a stable dispersion. Subsequently, 2.5 mL of glacial acetic acid was slowly added. The mixture was then transferred to an autoclave and then heated to 120 °C for 3 days. After the reaction, the product was collected by a magnet and washed several times with THF and methanol, and then dried at 60 °C under a vacuum.

### 3.5. MSPE Pretreatment

The prepared COF-SiO_2_@Fe_3_O_4_ nanocomposites were used to extract PYRs from an aqueous sample solution. Initially, 20 g of a crushed cabbage sample was placed into a centrifuge tube, and 20 mL of acetonitrile was added. The mixture was shaken for 20 min and then centrifuged at 4500 r·min^−1^ for 15 min to extract the supernatant. The supernatant was evaporated to about 3 mL. Then, it was further dried nearly to dryness using nitrogen gas. Subsequently, the dried residue was redissolved in ultrapure water to a final volume of 100 mL. Then, 10 mg of the COF-SiO_2_@Fe_3_O_4_ nanocomposite was dispersed in 20 mL of the sample solution under vigorous oscillation. The oscillation process lasted for 10 min to adsorb the target analytes until the adsorption equilibrium was achieved. Thereafter, the COF-SiO_2_@Fe_3_O_4_ nanocomposites with adsorbed PYRs were collected by using an external magnet and eluted with 1 mL acetone under 3 min of ultrasound. The desorption solution was collected, filtered through a 0.22 μm filter and injected for GC-MS analysis.

### 3.6. GC-MS Conditions

The GC was fitted with an Rxi-5si1MS column (30 m × 0.25 mm × 0.25 µm) (Shimadzu, Japan). Helium (99.999% purity) was utilized as the carrier gas. The inlet temperature was 250 °C and the sample was injected at 1 μL without splitting (1.08 mL·min^−1^). The initial oven temperature was controlled at 150 °C (held for 0.5 min), with a rate of 25 °C·min^−1^ to 180 °C and finally with a rate of 10 °C·min^−1^ to 250 °C (held for 8 min). The ion source temperature and interface temperature were 230 °C and 250 °C, respectively. The solvent delay was set to 2 min (bypassing the solvent peak). The separation of the PYRs was sufficient to set up the full scan in the range of 40–500 m/z. The selected ion monitoring (SIM) mode was used. Sample analysis was performed with the electron ionization source set at 70 eV. The mass spectral parameters of 5 PYRs (50 μg·L^−1^) are shown in Table 4.

### 3.7. Recovery

The recovery rate (ER) and enrichment factor (EF) were used to evaluate the extraction and enrichment abilities of the COF-SiO_2_@Fe_3_O_4_ magnetic nanoparticles on PYRs. The calculation formulas are as follows:

ER = (C_M_ × V_M_)/(C_0_ × V_aq_)

EF = C_M_/C_0_

where C_0_ is the concentration of PYRs added to the sample solution before magnetic solid-phase extraction, C_0_ = 50 μg·L^−1^. The concentration of PYRs in acetone elution after magnetic solid-phase extraction is denoted as C_M_. V_aq_ represents the volume of the sample solution before magnetic solid-phase extraction, V_aq_ = 20 mL. The volume of the acetone eluent after magnetic solid-phase extraction is represented by V_M_.

The experiments were repeated three times to validate the repeatability of the results. The results presented were the averages of the three experiments conducted. The Origin software was used as a statistical treatment to reach the conclusions described in the work.

## 4. Conclusions

In this research, magnetic nanocomposites of COF-SiO_2_@Fe_3_O_4_ were synthesized and utilized as adsorbents for the preconcentration of five PYRs via MSPE. The interaction between the benzene in COF-SiO_2_@Fe_3_O_4_ and the benzene of the analytes facilitated the effective extraction of PYRs from the sample solutions. The combination of COF-SiO_2_@Fe_3_O_4_-based MSPE with GC-MS resulted in the development of a fast, simple, highly efficient and sensitive method for the determination of trace PYRs, demonstrating a high enrichment factor, wide linear range, low detection limit and good reproducibility. Furthermore, the successful application in the selective enrichment and determination of trace PYRs in vegetables suggests that COF-SiO_2_@Fe_3_O_4_ nanocomposites have significant potential as novel adsorbents in sample pretreatment.

## Figures and Tables

**Table 1:** Linear ranges, regression equations, LODs and LOQs of five PYRs.

Analyte	Regression Equation	Linear Range /(μg⋅L^−1^)	r	LOD /(μg⋅kg^−1^)	LOQ /(μg⋅kg^−1^)	EF	RSD (%)						Batch to Batch (n = 3)
							Inter-Day (n = 5) Intra-Day (n = 6)						
							5	10	20	5	10	20	
Allethrin	y = 616.9x − 1255.5	5–100	0.9991	1.5	4.5	4.4	3.1	2.5	2.1	3.4	2.8	2.7	4.2
Tetramethrin	y = 780.02x + 754.75	5–100	0.9997	1.5	4.5	5.0	5.3	2.3	2.2	6.5	3.2	3.6	3.3
Bifenthrin	y = 4775.3x − 3182.1	1–100	0.9990	0.3	0.9	12.4	2.1	6.2	4.8	2.3	2.3	4.1	2.5
Fenpropathrin	y = 1077.2x + 195.48	2.5–100	0.9991	1.0	3.0	10.7	2.6	3.7	2.4	3.1	3.0	2.9	2.6
Cyhalothrin	y =451.16x − 561.9	5–100	0.9995	1.5	4.5	11.0	2.2	2.6	1.9	2.7	2.6	7.0	3.0

**Table 2:** Detection results of PYRs in three samples and spiked recoveries.

Analyte	Spiked Level /(μg⋅kg^−1^)	Cucumber Recovery (%, RSD%)	Cabbage Recovery (%, RSD%)	Lettuce Recovery (%, RSD%)
Allethrin	5	80.2 (3.5)	81.7 (3.1)	85.3 (7.0)
	10	90.1 (2.9)	91.3 (3.9)	87.3 (5.8)
	20	96.4 (3.4)	102.4 (4.1)	103.5 (6.1)
Tetramethrin	10	116.5 (6.1)	89.1 (5.3)	116.1 (4.5)
	20	97.2 (4.6)	107.6 (4.9)	110.2 (5.7)
	50	111.0 (6.1)	114.7 (5.7)	109.6 (4.8)
Bifenthrin	10	89.2 (5.4)	92.9 (2.1)	97.3 (3.6)
	20	96.6 (2.3)	95.3 (3.2)	94.2 (2.4)
	50	97.4 (3.9)	102.7 (4.2)	93.7 (3.4)
Fenpropathrin	10	112.5 (6.7)	107.5 (3.7)	116.7 (5.7)
	20	93.1 (5.8)	106.9 (5.6)	109.8 (3.2)
	50	107.8 (3.6)	105.2 (6.5)	107.1 (5.1)
Cyhalothrin	10	97.3 (2.9)	87.4 (6.8)	81.6 (6.5)
	20	89.9 (4.7)	108.5 (6.5)	89.3 (5.6)
	50	90.1 (5.1)	92.3 (5.3)	93.4 (5.4)

**Table 3:** Comparison of the proposed method with previous works.

Method	Samples	Number of Pyrethroids	Extraction Time (min)	Elution Solvent Consumption	LOD	Recovery (%)	Ref.
MSPE/GC-MS	environmental water samples	4	15	ethylacetate (2 mL) + acetone (0.5 mL)	0.05–0.21 μg⋅L^−1^	81.1–97.6%	[38]
SBSE/HPLC-UV	tobacco	6	50	acetonitrile (120 μL)	0.2–0.66 μg⋅L^−1^	82–117%	[10]
MSPE-LSE/GC-MS	fruit peels and fleshes	6	25	acetonitrile (2 mL)	0.04–0.14 μg⋅kg^−1^	73.6–123.1%	[39]
SPME/GC-μECD	fruit and vegetables	8	40		0.1–0.5 ng⋅g^−1^	88.5–103.5%	[11]
UADLLME/HPLC-UV	litchi fruit	6	13	chlorobenzene (310 μL) + methanol (100 μL)	1.15–2.46 μg⋅L^−1^	83.3–91.5%	[9]
MSPE/GC-MS	vegetables	5	10	acetone (1 mL)	0.3–1.5 μg⋅kg^−1^	80.2–116.7%	This work

**Table 4:** Retention times and qualitative and quantitative ions of 5 PYRs.

Peak Number	Compound	Retention Time (min)	Mass-to-Charge Ratio (*m*/*z*)	
			Quantitative Ion	Qualitative Ion
1	Allethrin	7.440	123	123, 79, 81
2	Tetramethrin 1	11.195	164	164, 123, 81
3	Bifenthrin	11.340	181	181, 166, 165
2	Tetramethrin 2	11.440	164	164, 123, 81
4	Fenpropathrin	11.670	97	97, 55, 181
5	Cyhalothrin	13.255	181	181, 197, 208

## Data Availability

The data are contained within the article.

## Supplementary Materials

The following supporting information can be downloaded at: https://www.mdpi.com/article/10.3390/molecules29102311/s1, Figure S1. Infrared spectra of COF-SiO_2_@Fe_3_O_4_, COF-SiO_2_@Fe_3_O_4_ immersed in acetone for 24 h and COF-SiO_2_@Fe_3_O_4_ as adsorbent after MSPE; Table S1. The basic properties of target PYRs.
