# Supplementary material for: COF-SiO2@Fe3O4 Composite for Magnetic Solid-Phase Extraction of Pyrethroid Pesticides in Vegetables
Source: Molecules. 2024 May 14;29(10):2311. doi: 10.3390/molecules29102311 (PMC11123868; doi:10.3390/molecules29102311)
Supplement: Supplementary file 1 [file molecules-29-02311-s001.zip › molecules-2887356-supplementary.pdf]

COF-SiO_2@Fe_3O_4 Composite for Magnetic Solid-Phase Extraction of Pyrethroid Pesticides in Vegetables

Ling Yu [1,2,*], Aiqing Xia [1], Yongchao Hao [1], Weitao Li [1], Xu He [1], Cuijuan Xing [1,*], Zan Shang [1] and Yiwei Zhang [1]

[1] College of Chemistry and Chemical Engineering, Xingtai University, Xingtai 054001, China; xiaaiqing59420@sina.com (A.X.); haoyongchao2015@163.com (Y.H.); liweitao2021xtxy@163.com (W.L.); hexunew@outlook.com (X.H.); s15530181703@163.com (Z.S.); zyw17659928836@163.com (Y.Z.)
[2] Functional Polymer Materials R&D and Engineering Application Technology Innovation Center of Hebei, Xingtai 054001, China
* Correspondence: 200820347@xttc.edu.cn (L.Y.); cuijuanxing@163.com (C.X.)

Table S1. The basic properties of target PYRs.

	Structure	Molecular weight	Kow
allethrin	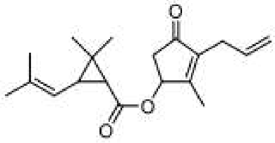	302.41	4.92
Tetramethrin	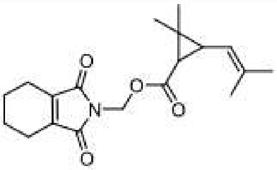	331.41	4.78
Bifenthrin	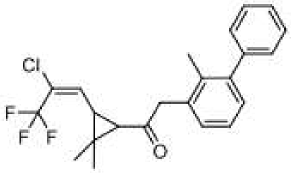	422.87	7.30
Fenpropathrin	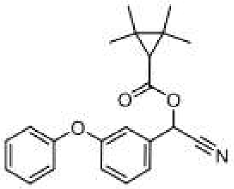	349.42	5.27
Cyhalothrin	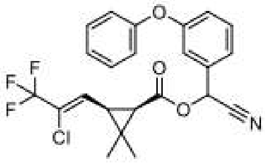	449.85	6.54

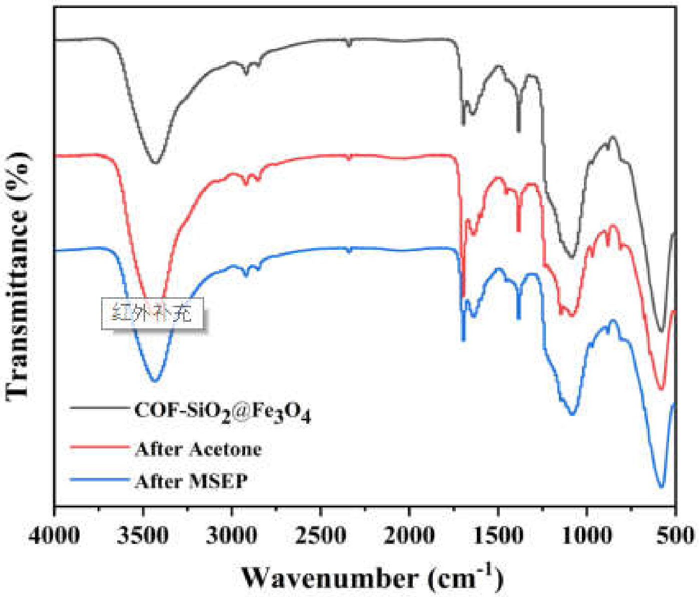

Figure S1. Infrared spectra of COF-SiO_2@Fe_3O_4 , COF-SiO_2@Fe_3O_4 immersed in acetone for 24 h and COF-SiO_2@Fe_3O_4 as adsorbent after MSPE.
